# Growth-rate regulated genes have profound impact on interpretation of transcriptome profiling in *Saccharomyces cerevisiae*

**DOI:** 10.1186/gb-2006-7-11-r107

**Published:** 2006-11-14

**Authors:** Birgitte Regenberg, Thomas Grotkjær, Ole Winther, Anders Fausbøll, Mats Åkesson, Christoffer Bro, Lars Kai Hansen, Søren Brunak, Jens Nielsen

**Affiliations:** 1 Institut für Molekulare Biowissenschaften, Johann Wolfgang Goethe-Universität, Max-von-Laue-Str. 9, 60438 Frankfurt am Main, Germany; 2 Center for Microbial Biotechnology, BioCentrum-DTU, Building 223, Technical University of Denmark, DK-2800 Kgs. Lyngby, Denmark; 3 Informatics and Mathematical Modelling, Building 321, Technical University of Denmark, DK-2800 Kgs. Lyngby, Denmark; 4 Center for Biological Sequence Analysis, BioCentrum-DTU, Building 208, Technical University of Denmark, DK-2800 Kgs. Lyngby, Denmark

## Abstract

**Background:**

Growth rate is central to the development of cells in all organisms. However, little is known about the impact of changing growth rates. We used continuous cultures to control growth rate and studied the transcriptional program of the model eukaryote *Saccharomyces cerevisiae*, with generation times varying between 2 and 35 hours.

**Results:**

A total of 5930 transcripts were identified at the different growth rates studied. Consensus clustering of these revealed that half of all yeast genes are affected by the specific growth rate, and that the changes are similar to those found when cells are exposed to different types of stress (>80% overlap). Genes with decreased transcript levels in response to faster growth are largely of unknown function (>50%) whereas genes with increased transcript levels are involved in macromolecular biosynthesis such as those that encode ribosomal proteins. This group also covers most targets of the transcriptional activator RAP1, which is also known to be involved in replication. A positive correlation between the location of replication origins and the location of growth-regulated genes suggests a role for replication in growth rate regulation.

**Conclusion:**

Our data show that the cellular growth rate has great influence on transcriptional regulation. This, in turn, implies that one should be cautious when comparing mutants with different growth rates. Our findings also indicate that much of the regulation is coordinated via the chromosomal location of the affected genes, which may be valuable information for the control of heterologous gene expression in metabolic engineering.

## Abstract 2

Analysis of *S. cerevisiae *cultures with generation times varying between 2 and 35 hours shows that the expression of half of all yeast genes is affected by the specific growth rate.

## Background

Growth is fundamental to proliferation of all living cells, from the most primitive prokaryote to human cells, and regulation of growth rate is essential if proper development of an organism is to take place. Despite progress in whole-genome transcription analysis [1,2], little is known about the transcriptional effects of differences in the growth rate, and most of this knowledge comes from indirect observations [3-5]. In many studies, cells treated with a metabolic inhibitor have a longer generation time [6,7]. This affects the expression of genes that encode ribosomal proteins (RPs) and enzymes involved in the central metabolism [7], but it is currently not possible, based on expression data alone, to distinguish between the primary effects caused by the addition of the metabolic inhibitor and the secondary effects arising from growth arrest. Likewise, transcription data from healthy mammalian tissue versus malignant tissue may be affected not only by the occurrence of specific mutations in the cancer cells but also by the difference in growth rate between the two types of tissue [8,9]. This hypothesis is substantiated by the finding that several hundred genes change expression level when comparing the slow-growing *Saccharomyces cerevisiae *mutant *mcm1 *with the corresponding wild-type strain, whereas very few genes change expression when the two strains are forced to grow with the same doubling time [10].

Here, we describe the transcriptional program over a wide range of doubling times in the yeast *S. cerevisiae *and discuss the implications for whole-genome transcriptome profiling. The growth rate of this lower eukaryote can be controlled in submerged, continuous culture by the feeding rate of nutrients. Cells grown in continuous culture at steady state have a specific growth rate, μ, that is equal to the dilution rate, defined as the ratio between the feeding rate and the volume of medium in the bioreactor. Because the specific growth rate is inversely proportional to the doubling time of the cells T_2 _(specifically, T_2 _= ln(2)/μ), it is possible to change the doubling times of cells in a controlled manner in continuous cultures. Although the environmental factors that control the specific growth rate in higher and lower eukaryotes are physiologically different, changes in the specific growth rate are expected to rely on the same basic biochemical changes. Comparative analysis of *Caenorhabditis elegans *and *S. cerevisiae *has also shown that most of the core biological functions are carried out by orthologous proteins [11], and the present study is therefore likely to reveal fundamental principles of growth control in eukaryotes.

## Results

### Consensus clustering reveals growth rate regulated genes

**Figure 1:**
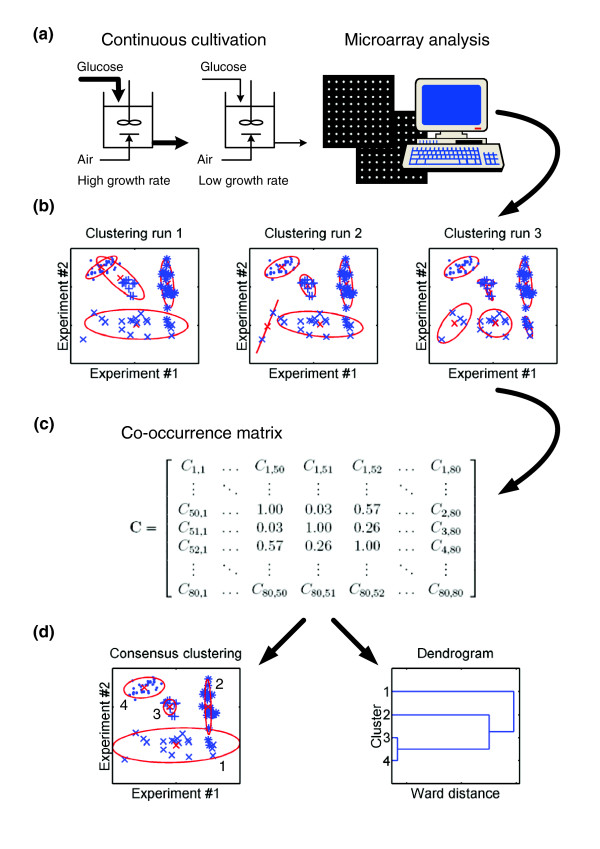
Experimental set-up. **(a) **Cells were grown at steady state in continuous chemostat cultures, with the specific growth rate controlled by the flow rate and the volume of medium in the reactor. Cells were harvested and used for transcription analysis and subsequent clustering of the transcription data. A simulated dataset was generated to illustrate the principles of consensus clustering. The dataset contained 80 members derived from four clusters (*, x, + and · in blue) in two experiments. The consensus clustering method consisted of three steps (panels b-d). **(b) **An ensemble of clusterings was obtained by multiple runs of mixture of Gaussians [59]. Each run gave very different results (red ellipses), depending upon the initialization. **(c) **The results from multiple runs was used to form the transcript co-occurrence matrix (*C*), which was calculated as the empirical probability (over all runs) of observing each pair of transcripts (*n*,*n'*) in the same cluster. **(d) **Based on the co-occurrence of transcripts a consensus clustering was generated. The co-occurrence matrix was also converted into a transcript-transcript distance matrix as *D*_*nn' *_= 1 - *C*_*nn'*_, which was used as input to a hierarchical clustering. The resulting consensus dendrogram showed the relationship between the clusters and was thereby a valuable tool in the biologic validation of the data.

The haploid laboratory strain *S. cerevisiae *CEN.PK113-7D was grown at steady state in aerobic chemostat cultures on a synthetic minimal medium with glucose as the limiting nutrient. Cells were cultured at six different specific growth rates, namely μ = 0.02, 0.05, 0.10, 0.20, 0.25, and 0.33 per hour, corresponding to doubling times between 2 and 35 hours (Figure 1a). To assess the transcriptional program underlying growth, we analyzed the whole-genome transcription profiles from all cultures and thereby identified a signal from 5,930 out of 6,091 annotated open reading frames (ORFs; Additional data file 1). The detectable transcripts were then grouped using a robust and signal insensitive algorithm for clustering of coexpressed genes, whereas genes with noisy expression profiles were discarded (Figure 1b-d) [12]. Consensus clustering algorithms [13-15] take advantage of the randomness in *K *means or Gaussian clustering solutions to produce a robust clustering. By averaging over multiple runs with different number of clusters *K*, common patterns in each clustering run are amplified whereas nonreproducible features of individual runs are suppressed. Consequently, it is possible to cluster large expression datasets without conservative fold change exclusion [12].

**Figure 2:**
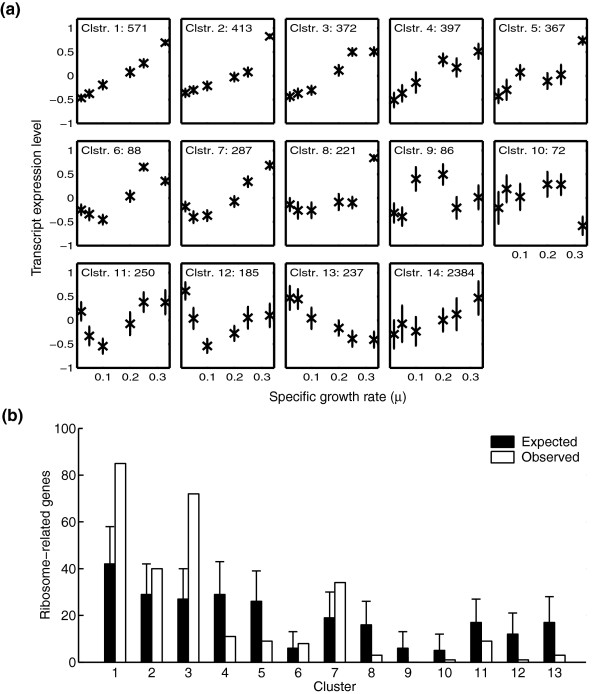
Clusters of genes that are coexpressed at specific growth rates from 0.02 to 0.33 per hour. **(a) **The transcript levels of differentially regulated genes are shown as transformed values between -1 and 1, where 0 indicates the average expression level over all six specific growth rates (μ = 0.02, 0.05, 0.1, 0.2, 0.25, and 0.33 per hour). The average transcript level within a cluster is indicated by the curve and the error bars give the standard deviation on the transcription profiles (clusters can be found in Additional data file 3). The 13 clusters originate from 27 clusters that were reduced manually (Additional data file 2). This was done by merging very similar clusters (clusters close in the dendrogram and discarding clusters that appeared to arise from experimental variation). Finally, ORFs that could not be assigned to a cluster with at least 80% probability (*P*_a _< 0.20) were discarded and collected into a 'trash' cluster 14 together with the discarded clusters. **(b) **shows the expected distribution of ribosome related genes (black bars) and the actual distribution of ribosome related genes (white bars) in the 13 clusters.

In the present case we extracted the consensus clusters from 50 scans with Gaussian mixtures in the interval *K *= 10 ... 40, leading to a total of 31 × 50 = 1,550 clustering runs. The results from the multiple runs were used to calculate a cooccurrence matrix *C*. This matrix describes the empirical probability of observing each pair of transcripts (*n*,*n'*) in the same cluster throughout the 1,550 clustering runs (Figure 1). The probability of transcript co-occurrence was then used to generate the consensus clusters (Additional data file 2). The co-occurrence matrix was converted into a transcript-transcript distance matrix as *D*_*nn' *_= 1 - *C*_*nn'*_; that is, a high probability of co-occurrence is equal to a short distance between the expression profiles of a pair of transcripts. The number of clusters in the dendrogram was finally determined as the average over the 50 repetitions of the Gaussian mixtures with the greatest likelihood. This criterion was found to be a pragmatic, conservative starting point for biologic validation. We reduced the 27 clusters to 13 by merging biologically similar clusters adjacent in the consensus dendrogram. Transcripts that could not be assigned to a cluster with at least 80% probability (*P*_a _< 0.20) were discarded and collected in a 'trash' cluster (Figure 2a, cluster 14; Additional data file 2).

### Transcript levels of genes involved in biogenesis increase with the specific growth rate

Among the 1753 ORFs (Figure 2a, clusters 1-4) with increasing transcript level as a function of the specific growth rate were mainly genes involved in RNA metabolism and in the biosynthesis of novel cell material. More specifically, these genes are involved in the synthesis of RPs, respiration, amino acid biosynthesis and lipid biosynthesis, as well as in nucleobase, nucleoside, nucleotide, and nucleic acid metabolism (Table 1). Ribosome-related genes were found to be over-represented in clusters 1, 3 and 7, and were almost absent in clusters with decreased or complex transcript patterns (Figure 2b). This observation was in good agreement with the over-representation of the regulatory ribosomal protein elements (RRPEs) GAAAA(A/T)TT in clusters 1 and 2 (Table 1). Comparing the genes of clusters 1-7 with a transcription factor binding study [16] showed that 70% of the RAP1 targets were found in these clusters, in particular clusters 2, 4, and 6 (*P *< 10^-2^). RAP1 is a highly abundant transcription factor [17] that is involved in transcriptional activation of the highly expressed genes, including genes encoding RPs and glycolytic enzymes [18]. The over-representation of RAP1 targets in clusters 2, 4, and 6 therefore suggests that this factor may be an important determinant of positive growth rate regulation.

A higher specific growth rate may be obtained by shortening steps in the cell cycle, and we therefore expected to identify cell cycle regulated genes among the growth rate affected genes [19]. Comparing a list of 430 cell cycle regulated genes [20-22] with genes regulated by the specific growth rate showed that this also was the case. Both clusters 1 and 2 exhibited significant over-representation of genes expressed in the G_1 _(*P *< 10^-2^) of the cell cycle. This observation, together with the finding of the M-G_1 _regulated RRPEs in genes of clusters 1 and 2, suggests that a change in the specific growth rate affected the length of G_1 _rather than other steps in the cell cycle.

### The transcript level of stress response genes decrease with the specific growth rate

**Figure 3:**
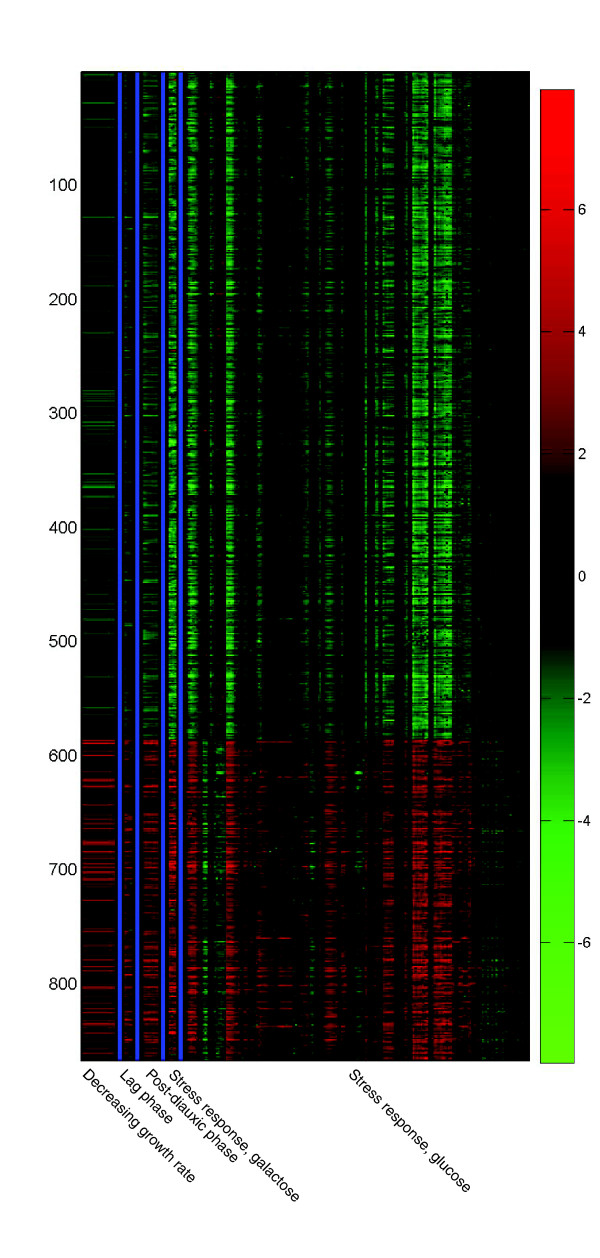
Comparison between conditions with changes in growth rate. From left to right separated by blue, vertical lines: the fold change in transcript levels between cells grown at lowest (average of μ = 0.02 and 0.05 per hour) and the highest growth rate (average of 0.33 per hour); cells in lag phase (four time points: 0, 0.01, 0.05, and 0.1 hours [5]); cells in postdiauxic phase (eight time points: 36, 51, 62, 83, 107, 130, 178, and 212.25 hours [5]); stress response, galactose (four time points: 20, 40, 60, and 140 min [6]); and ESR transcript profiles (right of blue vertical line) and 13 stress condition obtained from the work by Brown and coworkers (Figure 3 in their report [7]). The approximately 900 ESR genes were originally identified by hierarchical clustering of all yeast transcripts from 142 microarray experiments [7]. The transcripts formed two distinct clusters of transcript that responded similarly to 13 stress condition, and the corresponding genes were denoted the ESR genes [7]. Transcript levels from all conditions are based on a global normalization of the DNA arrays, in which it is assumed that the cellular mRNA levels remain constant in response to stress or changes in the specific growth rate (also see Additional data file 5). ESR, environmental stress response.

Many genes involved in stress response had decreased mRNA level as a function of the specific growth rate (Figure 2a, clusters 12 and 13). A signal that could be mediated by the TOR (target of rapamycin) pathway [23,24] via the corresponding stress response element, namely AGGGG, found to be over-represented among members of clusters 12 and 13 (Table 1). Genes in clusters 11 and 12 were mostly involved in chromosome organization and RNA processing, whereas cluster 13 typically contained stress response genes, for instance genes encoding heat shock proteins and genes involved in autophagy. To investigate the overlap between cluster 13 and genes found in stress response studies, we compared the present data with a core of 1,000 stress response genes that have been denoted the environmental stress response (ESR) genes [7]. Transcript data from cells going into lag phase [5], growing under postdiauxic conditions [5], or exposed to 12 stress conditions revealed a strong correlation with transcript profiles from cells at different specific growth rates (Figure 3). Eighty percent of the transcripts that decreased upon stress showed the same response to slower growth, whereas 89% of the transcripts that increased upon stress also increased upon slower growth (Figure 3). This overlap between growth rate regulated genes and genes responding to stress indicates that the stress response shares a component with the response to changes in the specific growth rate.

The analysis also revealed that the responses to stress and growth rate are independent of carbon source. Cells grown on galactose are inhibited when exposed to 10 mmol/l LiCl [25]. Besides a specific inhibition of phosphoglucomutase [25], lithium also inhibits the specific growth rate from 0.15 to 0.025 per hour over 140 minutes while the transcript level of 1,390 genes changed more than twofold [6]. The transcript profiles of these genes have a considerable overlap with those of glucose grown cells (Figure 3), and suggest that they relate to the growth rate rather than the choice and amount of carbon source.

Almost 50% of the members of cluster 13 (Figure 2) belonged to the group of ORFs with unknown process (Table 1). Overall, only 25% of the ORFs in *S. cerevisiae *have not been assigned to a biologic process, and the lack of annotation was therefore a clear trait of ORFs in cluster 13. The strong transcriptional response argued against these ORFs being dubious genes. Our results suggest that the cellular role played by these ORFs may be unclear because they are poorly expressed at the high specific growth rates at which phenotype and function are normally inferred.

### Ethanol production at high specific growth rates

Some clusters appeared bell or valley shaped, showing that many transcripts did not follow a simple dependence on the specific growth rate (Figure 2a, clusters 6 and 8-11). Genes in clusters 8 and 10 exhibited an abrupt change in transcript level at μ = 0.33 per hour, where the specific growth rate was above the so-called 'critical dilution rate' (μ = 0.30 per hour) at which the Crabtree effect sets in [26]. At this high specific growth rate the cells change from a respiratory metabolism to a mixed respiratory-fermentative metabolism, resulting in ethanol production (2.4 ± 0.1 g/l). The change in metabolism also correlated with induction of genes that are involved in vesicle transport and glucose transport (Figure 2a, cluster 8) and repression of genes that are involved in sporulation and carboxylic acid metabolism (Figure 2a, cluster 10). Most notable in the latter group were *ICL1 *and *MLS1*, which encode the key enzymes in the glyoxylate shunt; *ALD4 *and *ADH2*, which are involved in metabolism of ethanol; and *FBP1 *plus *PCK1*, which encode key gluconeogenic enzymes. *FBP1 *and *PCK1 *are previously reported to be subject to transcriptional repression at high glucose concentrations, although the mode of regulation is unclear because repression is not dependent on the *MIG1 *and Ras/cAMP pathways [27]. These observations suggested that increased glucose uptake, together with downregulation of genes that are involved in ethanol catabolism, gluconeogenesis, and the glyoxylate shunt, could be involved in a shift from pure respiratory metabolism to mixed respiratory-fermentative metabolism at high growth rates.

### Chromosomal organization of growth rate regulated genes

**Figure 4:**
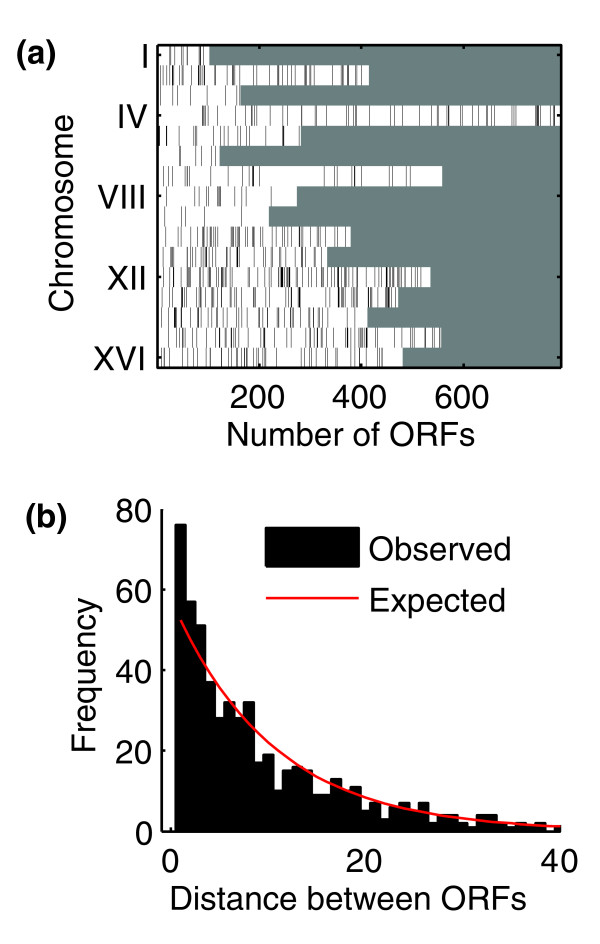
Chromosomal position of the genes in cluster 1. Shown are genes at **(a) **the chromosomal level and **(b) **at the local level between ORFs. The 16 chromosomes in panel **(a) **are shown in white and cluster members as vertical black bars on the chromosomes. The length of the chromosomes are scaled according to the number of ORFs on a given chromosome. (**b**) The distance between ORFs from cluster 1 (x-axis) measured in number of ORFs. The expected distance is shown with a red curve while the actual distance between ORFs is shown with black bars. ORF, open reading frame.

The cluster analysis also revealed that gene pairs had much greater probability of being coexpressed than would be expected if they were randomly distributed across the genome (Figure 4a,b). The exception to this pattern was genes in one of the upregulated clusters and genes that changed expression abruptly around the critical dilution rate of μ = 0.30 per hour (clusters 1, 8, and 10); otherwise, all other clusters had an over-representation of gene pairs or genes in close vicinity to each other on the chromosomes.

Short chromosomal domains of coexpressed genes have previously been reported for *S. cerevisiae *and the *Drosophila *genome [28,29]. It has been suggested that gene expression within a chromosomal domain behaves as a 'square wave' (a discrete opening of the chromatin gives the transcriptional machinery increased access to several neighboring promoters) [29,30]. Opening of the chromatin occurs when the nucleosomes are remodeled by factors such as RAP1 [31] and during DNA replication. We therefore speculated that the coexpression of growth-rate regulated genes (Figure 4a,b) could be influenced by replication and tested if there was a significant over-representation of these genes around the replication origins. In *S. cerevisiae*, 429 replication origins have been determined by chromosome immunoprecipitation [32] and 332 origins have been found by replication timing experiments [33]. Between these two sets, 294 replication origins were overlapping within 10 kilobases (kb) [34].

**Figure 5:**
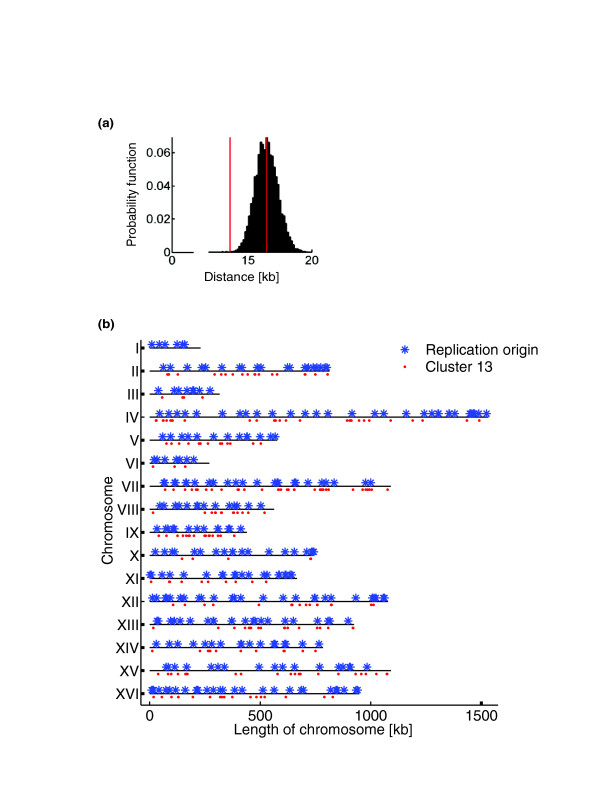
Chromosomal location of replication origins (blue replication origins) and ORFs from cluster 13 (red dots). A randomization test revealed that the average ORFs are much closer to the replication origins than would be expected by chance. **(a) **The actual and expected average distance between ORFs and replication origins are shown with red lines to the left and right, respectively. The variation of the expected distance is indicated with a black histogram. **(b) **The genomic position of genes in cluster 13 (red dots) and replication origins (blue stars).

Comparing the chromosomal position of the growth-related genes in clusters 1-13 (Figure 2) with the 294 replication origins revealed a positive correlation (*P *< 10^-3^) between the genes and distance to the nearest replication origins. The average distance for a gene in these clusters to the nearest replication origins was 16.41 kb, whereas the average distance expected by chance was 16.81 ± 0.15 kb (average/standard deviation). Within the group of growth-regulated genes it was observed that genes in downregulated cluster 13 were found to be positioned closer to the replication origins than would be expected by chance (Figure 5). The average distance for a gene in cluster 13 to the nearest replication origins was 13.57 kb, whereas the average distance expected by chance was 16.43 ± 0.88 kb (average/standard deviation; *P *< 10^-3^). One explanation for this phenomenon could be that some of the genes in cluster 13 are direct neighbors to the replication origins, whereas the remaining ones are distributed on the chromosomes as would be expected based on chance. Because of the correlation between transcript profiles from different growth rates and stress conditions (Figure 3), we speculated that genes responding to stress, postdiauxic shift, and stationary phase would also be closer to origins than expected by chance (see Table S5 in the report by Radonjic and coworkers [5], published elsewhere). Interestingly, this appeared to be the case for genes with altered expression in response to the stationary phase after diauxic shift (see Table S5 in the report by Radonjic and coworkers [5], published elsewhere). The average distance of the upregulated genes was 15.27 kb whereas the average distance expected by chance was 16.81 ± 0.65 kb (*P *< 10^-2^). If growth-regulated genes are closer to the replication origins, then it would be expected that non-growth regulated genes are further away from the replication origins. This indeed was also the case when comparing the genes with marginal changes in expression under different growth conditions (see cluster F in Figure 3 in the report by Radonjic and coworkers [5], published elsewhere) to the position of the replication origins (*P *< 10^-3^).

We also included a sensitivity analysis to evaluate the influence of the number of replication origins used in the analysis. The sensitivity analysis showed that the *P *values decreased with increasing number of replication origins (Additional data file 4). The number of replication origins is based on two datasets including 429 and 332 origins. Thus, the true number of replication origins is expected to be higher than 294. If the true number of replication origins is higher then the *P *values in the analysis are very conservative, and this would add further confirmation of our conclusions.

## Discussion

The present study shows that changes in specific growth rate have profound and complex effects on gene expression in *S. cerevisiae*. One of the clearest traits in the dataset is the gradual upregulation of RP genes in response to higher specific growth rates (Figure 2a and Table 1), and downregulation of genes with the stress response element in their promoter. The opposite effect is often found in transcription studies, where the effects of stress are investigated. Exposure of yeast cells to seven types of stress [35], 11 environmental changes [7], lithium [6], rapamycin [36], or the GCN pathway inducer 3-aminotriazole [37] led to reduced expression of RP genes and induction of STRE genes covering a core of 1,000 ESR genes [7]. The data presented here reveal that almost all ESR genes respond similarly to stress and decreased growth rate. Because conditions known to induce ESR genes often inhibit growth [6,7,35], it is tempting to speculate that the growth rate response and the stress response are regulated by a common component. A similar phenomenon has been reported for *Escherichia coli*, for which the specific growth rate is known to control the general stress response via the concentration of the general stress response sigma factor RpoS [38].

In addition to the ESR genes, we found that another 2,000 genes were affected by changes in the specific growth rate. These transcripts may witness a second slow response to changes in the specific growth rate. Our experiments were conducted in cells that had reached a physiologic steady state, which was defined as five generations of growth without changes in the measured biomass concentration, pH, carbon dioxide, and oxygen values. The cells may thereby both go through a rapid response to changes in the specific growth rate, which simulates the stress response, and a slow response that enables prolonged survival at a given specific growth rate.

Besides specific transcription factors, chromosome organization may also contribute to the regulation of the growth rate regulated genes. This includes a location adjacent to the replication origins, as well as over-representation of coexpressed gene pairs. These modes of regulation have until recently been given little attention, because the gene order in the eukaryotic cell has mostly appeared random compared with the highly organized, polycistronic structures in bacteria [39]. This view has changed as whole-genome studies have shown that some coregulated genes are colocated in the chromatin, such as the yeast cell cycle regulated genes, in which genes in the same phase are found to colocate in the chromatin [20,28]. In yeast coregulated genes tend to be spaced in a periodic pattern along the chromosome arms [40], supporting the view that higher order chromatin structures could play a role in gene expression. Coexpression of gene pairs can to some extent be explained by bidirectional promoters [20,28]. However, convergent gene pairs, tandem pairs, and longer stretches cannot be regulated by this mechanism [20,28,41] but must be controlled at a higher level such as by histone modifications. Candidates are histone acetylation patterns that are known to correlate with blocks of coexpressed genes [42].

Histone modifications may also explain the co-occurrence of replication origins and growth rate regulated genes. Histones are removed from the chromatin by chromatin remodeling factors (for example, RAP1 [31]), which open the chromatin for transcription [43] as well as replication [44]. We found that most RAP1 targets are positively regulated by growth rate. In accordance with this observation and the role of RAP1 in replication, we also found growth rate regulated genes to be located closer to the replication origins than would be expected by chance (Figure 5). A signal for chromatin remodeling could be mediated by histone acetylation. Deletion of the histone deacetylase gene, *RPD3*, has a positive effect on both replication and transcription [45,46]. Acetylation of histones around the replication origins leads to early replication in the S phase [46]. Early replication [47] as well as RPD3 location are again known to correlate with high gene expression [48,49]. We therefore propose a model in which the histone modifications around the replication origins change as a function of the specific growth rate and thereby confer transcriptional changes to the adjacent genes.

A caveat of our analysis is the fact that by using glucose limiting cultures to control the specific growth rate, we also slightly vary the glucose concentration in the medium. Part of our findings may therefore be explained by the change in glucose concentration. However, as most of our experiments were carried out below the critical dilution rate (μ = 0.30 per hour), at which the glucose concentration is too low to cause repression (< 0.02 g/l), we are confident that the majority of the observed effects are caused by the variation in the specific growth rate. Four facts support our contention that the major variant in the experiments is the growth rate. First, we identified RP genes, which are known to be induced under growth via the growth-regulating TOR pathway [50]. Second, none of the known consensus elements for glucose repression/induction were over-represented among genes with a positive transcript profile, as would be expected if glucose should affect expression below the critical dilution rate. This pertains to *MIG1 *and *RGT1*, as well as to the *HAP2*/*3*/*4*/*5 *binding sites. Third, only 117 genes exhibited a significant change in transcript level when sugars (glucose and maltose) where compared with C2 compounds (acetate and ethanol) in aerobic continuous cultivations at one specific growth rate [51]. Finally, we found almost complete overlap in affected genes between the current data and data from cells changing growth rate on the nonrepressive carbon source galactose (Figure 3).

## Conclusion

We found that changing specific growth rates has a substantial impact on transcript levels in the eukaryotic model *S. cerevisiae*. Varying the doubling time between 2 and 35 hours affects the expression of half of the genes in the genome, including most of the genes affected by stress. This finding suggests that the growth rate may play a role in stress response and that caution should be exercised when transcript data from cells under stress or mutants with different growth rates are compared. Much of the transcriptional regulation may be mediated via RAP1, the RRPE, and the stress response element in promoters of the affected genes. Moreover, other effects such as coexpression of neighbouring genes and the location of many genes adjacent to replication origins also appear to play a role in regulation.

## Materials and methods

### Strain and continuous cultivations of *S. cerevisiae*

CEN.PK113-7D *MAT*a was grown at dilution rates of 0.02, 0.05, 0.10 (in triplicate), 0.20 (in triplicate), 0.25, and 0.33 (in triplicate) per hour. The strain background and the aerobic continuous cultivations were described previously [52,53].

### DNA microarray analysis and data acquisition

The cRNA synthesis, hybridization to Affymetrix S98 arrays, and scanning were performed as described previously [54] with the only exception that the hybridization signal was not amplified, because we found that this step conferred substantial noise on the expression data. Affymetrix Microarray Suite v5.0 (Affymetrix Inc., Santa Clara, CA, USA) was used to generate CEL files of the scanned DNA microarrays. The normalized expression levels of the 9335 probe sets were subsequently calculated using the Perfect Match model in dChip v1.2 [55], and this dataset was used to extract the expression level of 6091 annotated unique ORFs (updated March, 2004) [56]. The data have been deposited at ArrayExpress [57] with the accession number E-MEXP-593.

### Normalization

To compensate for a drop in the mRNA level at different growth rates [58], we identified 42 ORFs that decreased linearly with specific growth rate (*P *< 0.05) with an average ratio of 1.8, and we used this information to scale the dataset such that the 42 selected ORFs had constant expression for all specific growth rates (Additional data files 1 and 5).

### Consensus cluster analysis

For all experiments done in triplicates, the geometric average was calculated as follows:

Y=[∏m=13Ym]1/3
 MathType@MTEF@5@5@+=feaafiart1ev1aaatCvAUfeBSjuyZL2yd9gzLbvyNv2Caerbhv2BYDwAHbqedmvETj2BSbqee0evGueE0jxyaibaiKI8=vI8tuQ8FMI8Gi=hEeeu0xXdbba9frFj0=OqFfea0dXdd9vqai=hGuQ8kuc9pgc9s8qqaq=dirpe0xb9q8qiLsFr0=vr0=vr0dc8meaabaqaciGacaGaaeqabaqadeqadaaakeaacaWGzbGaeyypa0ZaamWaaeaadaqeWbqaaiaadMfadaWgaaWcbaGaamyBaaqabaaabaGaamyBaiabg2da9iaaigdaaeaacaaIZaaaniabg+GivdaakiaawUfacaGLDbaadaahaaWcbeqaaiaaigdacaGGVaGaaG4maaaaaaa@40F7@

The transformed expression level (*n *= 1 ... *N *transcript index, and *m *= 1 ... *M *chip index) was used for visualization:

Xnm=(Ynm−Y¯n)/∑m=1M(Ynm−Y¯n)2
 MathType@MTEF@5@5@+=feaafiart1ev1aaatCvAUfeBSjuyZL2yd9gzLbvyNv2Caerbhv2BYDwAHbqedmvETj2BSbqee0evGueE0jxyaibaiKI8=vI8tuQ8FMI8Gi=hEeeu0xXdbba9frFj0=OqFfea0dXdd9vqai=hGuQ8kuc9pgc9s8qqaq=dirpe0xb9q8qiLsFr0=vr0=vr0dc8meaabaqaciGacaGaaeqabaqadeqadaaakeaacaWGybWaaSbaaSqaaiaad6gacaWGTbaabeaakiabg2da9iaacIcacaWGzbWaaSbaaSqaaiaad6gacaWGTbaabeaakiabgkHiTiqadMfagaqeamaaBaaaleaacaWGUbaabeaakiaacMcacaGGVaWaaOaaaeaadaaeWbqaaiaacIcacaWGzbWaaSbaaSqaaiaad6gacaWGTbaabeaakiabgkHiTiqadMfagaqeamaaBaaaleaacaWGUbaabeaakiaacMcadaahaaWcbeqaaiaaikdaaaaabaGaamyBaiabg2da9iaaigdaaeaacaWGnbaaniabggHiLdaaleqaaaaa@4D5A@

Here Y¯n
 MathType@MTEF@5@5@+=feaafiart1ev1aaatCvAUfeBSjuyZL2yd9gzLbvyNv2Caerbhv2BYDwAHbqedmvETj2BSbqee0evGueE0jxyaibaiKI8=vI8tuQ8FMI8Gi=hEeeu0xXdbba9frFj0=OqFfea0dXdd9vqai=hGuQ8kuc9pgc9s8qqaq=dirpe0xb9q8qiLsFr0=vr0=vr0dc8meaabaqaciGacaGaaeqabaqadeqadaaakeaaceWGzbGbaebadaWgaaWcbaGaamOBaaqabaaaaa@3542@ is the average expression level for the *n*th transcript and the denominator is the Euclidean norm over the *M *experiments. Hence, the transformed transcript level *Xnm *is confined to the interval [-1,1]. A value of 0 corresponds to the mean average level over all six specific growth rates. The dataset was clustered *R *= 31 × 50 = 1,550 times, *K *= 10 ... 40 clusters and 50 repetitions for each size, with the variational Bayes mixture of Gaussians [59]. For each run *r *this gave a cluster label matrix label(*n*,*r*), along with a likelihood, which was used to calculate the co-occurrence matrix *C*_*nn' *_(i.e. the empirical probability that two transcripts *n *and *n' *were in the same cluster).

Cnn′=1R∑r=1Rδ(label(n,r),label(n′,r))
 MathType@MTEF@5@5@+=feaafiart1ev1aaatCvAUfeBSjuyZL2yd9gzLbvyNv2Caerbhv2BYDwAHbqedmvETj2BSbqee0evGueE0jxyaibaiKI8=vI8tuQ8FMI8Gi=hEeeu0xXdbba9frFj0=OqFfea0dXdd9vqai=hGuQ8kuc9pgc9s8qqaq=dirpe0xb9q8qiLsFr0=vr0=vr0dc8meaabaqaciGacaGaaeqabaqadeqadaaakeaacaWGdbWaaSbaaSqaaiaad6gaceWGUbGbauaaaeqaaOGaeyypa0ZaaSaaaeaacaaIXaaabaGaamOuaaaadaaeWbqaaiabes7aKbWcbaGaamOCaiabg2da9iaaigdaaeaacaWGsbaaniabggHiLdGcdaqadaqaaiaadYgacaWGHbGaamOyaiaadwgacaWGSbGaaiikaiaad6gacaGGSaGaamOCaiaacMcacaGGSaGaamiBaiaadggacaWGIbGaamyzaiaadYgacaGGOaGabmOBayaafaGaaiilaiaadkhacaGGPaaacaGLOaGaayzkaaaaaa@53A2@

where δ (*l*,*l'*) = 1 if *l *= *l'*, and δ (*l*,*l'*) = 0 otherwise [13-15]. Contrary to a distance matrix calculated directly in 'expression level space', the 'consensus distance' *D*_*nn' *_= 1 - *C*_*nn' *_was not suffering from outlier effects. Thus, based on the consensus distance, data could be clustered reliably with hierarchical clustering using the Ward algorithm (Additional data files 2 and 3). Second, the likelihood was used to estimate the initial number of clusters to 27 (number of leaves in the hierarchical clustering). A thorough description of the cluster algorithm and the biological validation for reducing the number of clusters to 13 can be found in Additional data file 2 and in the report by Grotkjær and coworkers [12].

### Statistical tests

The expected distance between two coexpressed genes was calculated by assuming that a given gene belongs to a given cluster with probability *P *= *Z/N*. Here, *Z *is the number of transcripts in the analyzed cluster, and *N *denotes the total number of transcripts in the DNA microarray analysis found in the systematic sequence of S288C (6081). The distance between two genes belonging to the same cluster follows the negative binomial distribution (*r *= 1, *P *= *Z*/*N*). *Z *genes distributed on 16 chromosomes give rise to (*Z *- 16) intervals between genes. Hence, the expected number of times, *Z*_*D*_, the distance *D *between two co-expressed genes is encountered is as follows:

ZD=(Z−16)ZN(1−ZN)D
 MathType@MTEF@5@5@+=feaafiart1ev1aaatCvAUfeBSjuyZL2yd9gzLbvyNv2Caerbhv2BYDwAHbqedmvETj2BSbqee0evGueE0jxyaibaiKI8=vI8tuQ8FMI8Gi=hEeeu0xXdbba9frFj0=OqFfea0dXdd9vqai=hGuQ8kuc9pgc9s8qqaq=dirpe0xb9q8qiLsFr0=vr0=vr0dc8meaabaqaciGacaGaaeqabaqadeqadaaakeaacaWGAbWaaSbaaSqaaiaadseaaeqaaOGaeyypa0JaaiikaiaadQfacqGHsislcaaIXaGaaGOnaiaacMcadaWcaaqaaiaadQfaaeaacaWGobaaamaabmaabaGaaGymaiabgkHiTmaalaaabaGaamOwaaqaaiaad6eaaaaacaGLOaGaayzkaaWaaWbaaSqabeaacaWGebaaaaaa@425C@

The statistical significance between the position of replication origins and ORFs in each cluster was determined by randomization tests. For all genes in a particular cluster, the average distance between the start codon in base pairs to the nearest of the 294 replication origins [34] was calculated. The average distance for clusters with genes evenly distributed over all chromosomes was repeatedly determined, and a *P *value (the probability for observing the average distance in the cluster by chance) was calculated. The number of replication origins used in this study is less than the 429 replication origins determined by chromosome immunoprecipitation [32] and 332 found by replication timing experiment [33]. A sensitivity analysis revealed that the *P *value increased for less than 294 replication origins and so the calculated *P *values should be considered conservative estimates.

The cumulated hypergeometric distribution was used to test for over-representation of cluster members among both cell cycle regulated genes and the transcription factor RAP1.

P=1−∑i=0X−1(Ki)(N−KZ−i)(NZ)
 MathType@MTEF@5@5@+=feaafiart1ev1aaatCvAUfeBSjuyZL2yd9gzLbvyNv2Caerbhv2BYDwAHbqedmvETj2BSbqee0evGueE0jxyaibaiKI8=vI8tuQ8FMI8Gi=hEeeu0xXdbba9frFj0=OqFfea0dXdd9vqai=hGuQ8kuc9pgc9s8qqaq=dirpe0xb9q8qiLsFr0=vr0=vr0dc8meaabaqaciGacaGaaeqabaqadeqadaaakeaacaWGqbGaeyypa0JaaGymaiabgkHiTmaaqahabaWaaSaaaeaadaqadaqaauaabeqaceaaaeaacaWGlbaabaGaamyAaaaaaiaawIcacaGLPaaadaqadaqaauaabeqaceaaaeaacaWGobGaeyOeI0Iaam4saaqaaiaadQfacqGHsislcaWGPbaaaaGaayjkaiaawMcaaaqaamaabmaabaqbaeqabiqaaaqaaiaad6eaaeaacaWGAbaaaaGaayjkaiaawMcaaaaaaSqaaiaadMgacqGH9aqpcaaIWaaabaGaamiwaiabgkHiTiaaigdaa0GaeyyeIuoaaaa@4BB1@

Here, *X *is the number of transcripts in each phase of the cell cycle found by the cluster analysis and *K *is the total number of analyzed ORFs in each phase of the cell cycle. *N *and *Z *are defined as above. We tested over-representation and under-representation of all 14 clusters in each phase of the cell cycle, and corrected the *P *value for multiple testing [60], leading to a cut-off of *P *< 0.01. Cell cycle regulated genes were compiled by selecting genes appearing in at least two of four lists, one containing genes known to be involved in the cell cycle based on literature studies and three lists arising from independent, numerical analyses [20-22]. A list of 5,421 overlapping genes was compiled by comparing the current dataset with that reported in the transcription factor binding study conducted by Lee and coworkers [16]. The transcription factor RAP1 was found to affect 288 genes (*P *< 0.01). The genes were distributed in the clusters as follows: clusters 1-7 contained 132 genes, the 'trash' cluster 101 genes, and other clusters 55 genes.

## Additional data files

The following additional data are available with the online version of this paper. Additional data file 1 is a table showing the expression profiles (all specific growth rates) of the 6,091 annotated unique ORFs (including 'not physically mapped' and 'not in systematic sequence of S288C' ORFs) from the *Saccharomyces *Genome Database [56] (updated March 2004). Additional data file 2 is a document describing the principles of the robust clustering method based on a Bayesian consensus mechanism. Additional data file 3 is a document including results of the cluster analysis. Additional data file 4 is a document showing the influence of the number of replication origins on the *P *values when testing for correlation between genes and their location with respect to the replication origins. Additional data file 5 is a document describing the normalization with dChip and the subsequent comparison with a whole genome study with external RNA control as normalization reference.

## Supplementary Material

Additional data file 1 The expression profiles (all specific growth rates) of the 6,091 annotated unique open reading frames (ORFs; including 'not physically mapped' and 'not in systematic sequence of S288C' ORFs) from the *Saccharomyces *Genome Database [56] (updated March 2004) can be viewed. Each gene can be selected by its name or, in case the gene has not been named, by its corresponding ORF name. Click here for file

Additional data file 2 Document describing the principles of the robust clustering method based on a Bayesian consensus mechanism. Click here for file

Additional data file 3 Document including results of the cluster analysis. Click here for file

Additional data file 4 Document showing the influence of the number of replication origins on the *P *values when testing for correlation between genes and their location with respect to the replication origins. Click here for file

Additional data file 5 Document describing the normalization with dChip and the subsequent comparison with a whole genome study with external RNA control as normalization reference. Click here for file

## Figures and Tables

**Table 1:** Over-represented GO groups and promoter consensus sequences

Cluster	GO group
Cluster 1	Metabolism
	Biosynthesis
	Cell organization and biogenesis
	Amino acid metabolism
	Nucleotide metabolism
	Protein metabolism
	Nucleotide biosynthesis
	Carboxylic acid metabolism
	tRNA modification
	Ribosome biogenesis and assembly
	Nucleobase, nucleoside, nucleotide, and nucleic acid metabolism
	Glutamate biosynthesis
	TGAAAA/TTTTCA
	GAAAAA/TTTTTC

Cluster 2	Cell growth and/or maintenance
	Mitotic cell cycle
	Physiologic process
	Nuclear organization and biogenesis
	Organelle organization and biogenesis
	Cytoplasm organization and biogenesis
	Cytoskeleton organization and biogenesis
	Morphogenesis
	Reproduction
	AAATTT/AAATTT
	GAAAAA/TTTTTC

Cluster 3	Ribosome biogenesis
	Cytoplasm organization and biogenesis
	RNA metabolism
	Aerobic respiration
	Nucleobase, nucleoside, nucleotide, and nucleic acid metabolism
	Cell growth and/or maintenance
	AATTCA/TGAATT

Cluster 4	Lipid metabolism
	Steroid metabolism
	Amino acid biosynthesis
	Glutamine family amino acid biosynthesis
	Cell growth and/or maintenance
	Arginine biosynthesis
	ATAACA/TGTTAT

Cluster 5	Cell growth and/or maintenance
	Protein modification
	Protein amino acid phosphorylation
	Organelle organisation and biogenesis
	Cell wall organization and biogenesis
	Cell organization and biogenesis
	Signal transduction
	Cytokinesis
	Amino acid biosynthesis

Cluster 6	DNA replication and chromosome cycle

Cluster 7	-

Cluster 8	Transport
	GAAAAA/TTTTTC

Cluster 9	Steroid metabolism
	Alcohol metabolism
	Ergosterol biosynthesis
	Ammonium transport
	Biological process unknown

Cluster 10	Carboxylic acid metabolism
	Sporulation
	Nitrogen utilization
	Carnitine metabolism
	Main pathways of carbohydrate metabolism
	Energy pathways
	Sporulation

Cluster 11	RNA splicing
	mRNA metabolism
	Regulation of transcription

Cluster 12	Meiosis
	Meiotic prophase I
	Nuclear division
	Response to stimulus
	AAGGGG/CCCCTT

Cluster 13	Autophagy
	Vitamin metabolism
	Fatty acid β-oxidation
	Response to water
	Biological process unknown
	AAGGGG/CCCCTT
	AGGGAG/CTCCCT
	AAAAGG/CCTTTT
	AAAGGG/CCCTTT
	AGGGGG/CCCCCT

Shown are over-represented GO [61,62] groups and promoter consensus sequences in the 13 clusters of growth regulated genes. GO groups describing a cellular process with *P *< 10^-4 ^were considered significant and included in the table. If the same set of genes was found in two or more neighbouring GO groups, only one GO term is included [63]. Hexamers, found in the 800 base pair upstream region of ORFs in a cluster, were considered significantly over-represented when *E *< 10^-2 ^[64,65]. GO, Gene Ontology; ORF, open reading frame.
