# Supplementary material for: Growth-rate regulated genes have profound impact on interpretation of transcriptome profiling in Saccharomyces cerevisiae
Source: Genome Biol. 2006 Nov 14;7(11):R107. doi: 10.1186/gb-2006-7-11-r107 (PMC1794586; doi:10.1186/gb-2006-7-11-r107)
Supplement: Additional data file 2 — Document describing the principles of the robust clustering method based on a Bayesian consensus mechanism. [file gb-2006-7-11-r107-S2.pdf]

Additional data file 2

In this additional data file we describe the principles of the applied clustering method. A thorough description of the algorithm can be found in Grotkjaer et al. [12].

In the *first* step, all 5,930 detectable *Saccharomyces* Genome Database (SGD) annotated ORFs were clustered with a robust clustering method based on a Bayesian consensus mechanism. In the *second, interactive* step, we manually removed clusters arising from experimental artefacts and merged clusters representing the same biological information. Here we briefly review the initial multiple clustering and then we describe the robust consensus clustering. Finally, in the *third* step, we removed ORFs that were not assigned to any cluster with at least 80% probability, i.e. $P_a < 0.20$. This P_a-value was based on one clustering run, in which the VBMoG clustering run was initiated in the consensus solution of the merged clusters (clusters representing artefacts were removed).

Multiple clustering

We first clustered the transcription data x_{nm}, where $n = 1, \ldots, N$ is the transcript (5,930 in total) and $m = 1, \ldots, M$ is the specific growth rate (6 in total) as a mixture of K Gaussians (MoG)

$$p(\mathbf{x}) = \sum_{k=1}^{K} p(k)p(\mathbf{x}|k) \ , \tag{1}$$

where $\mathbf{x} = (x_1, \ldots, x_M)$ are M-dimensional data vectors, corresponding to one transcript at 6 different specific growth rates. $p(k)$ is the mixing proportions (which sum to one: $\sum_k p(k) = 1$) and $p(\mathbf{x}|k)$ is a Gaussian with mean vector $\boldsymbol{\mu}_k$ and covariance $\boldsymbol{\Sigma}_k$

$$p(\mathbf{x}|k) = \frac{1}{\sqrt{(2\pi)^M \det \Sigma_k}} \exp\left(-\frac{1}{2}(\mathbf{x} - \boldsymbol{\mu}_k)^T \boldsymbol{\Sigma}_k^{-1}(\mathbf{x} - \boldsymbol{\mu}_k)\right) \ . \tag{2}$$

In the variational Bayes (VB) approach, transcripts are first assigned to random clusters, and then the maximum likelihood is found by a number of iterative expectation maximisation steps which converge to a maximum of the likelihood [59]. The responsibility $p(k|\mathbf{x}_n)$, which is the probability of cluster k given transcript n, was used to assign the transcript to the most probable cluster $k = 1, \ldots, K$.

$$\text{assignment}(n) = \underset{k}{\text{argmax}}\, p(k|\mathbf{x}_n) \ . \tag{3}$$

Thus, the responsibility matrix gave us the assignment of each transcript to each Gaussian, e.g. for $K = 3$ we could have a transcript n with $p(k|\mathbf{x}_n) = (0.95, 0.01, 0.04)$ corresponding to a case with soft assignments. If the transcript were assigned to one and only one Gaussian we had hard assignments, e.g. $p(k|\mathbf{x}_n) = (1, 0, 0)$. Secondly, the procedure was repeated with the consensus clustering assignments instead of random initialisation. If $\max p(k|\mathbf{x}_n) > 0.80$, which we define as a cluster assignment P_a-value $P_a < 0.20$, the transcript was included in the analysis. This P_a-value is arbitrary and not conservative, but it was found by trial-and-error that it gave the best and clearest interpretation of the transcription data.

Robust consensus clustering

As mentioned in the article we extracted the robust consensus clustering from mixture of Gaussians clustering runs with $K = 10, \ldots, 40$ clusters and 50 repetitions for each size. This led to $R = 31 \cdot 50 = 1,550$ runs. A single run r gave a responsibility matrix $p(k|\mathbf{x}_n, r)$, and from this quantity we calculated the *co-occurrence matrix* in **Equation 4** (dimensions

$5,930 \times 5,930$), which was the probability that two different transcripts n and n', i.e. ($n \neq n'$), are in the same cluster.

$$C_{nn'} = \sum_{r=1}^{R} \sum_{k=1}^{K_r} p(k|\mathbf{x}_n, r) p(k|\mathbf{x}_{n'}, r) \ . \tag{4}$$

The *co-occurrence matrix* was determined by summing over all runs and normalising all values in C to values between 0 and 1. In the extreme cases $C_{nn'} = 0$ the transcripts never fell in the same clusters while $C_{nn'} = 1$ showed that transcripts always fell in the same clusters. Note, if we used hard assignments, i.e. $p(k|\mathbf{x}_n, r)$ was either zero or one (and sums to one), then **Equation 4** could be simplified to the form

$$C_{nn'} = \frac{1}{R} \sum_{r=1}^{R} \delta(c(n, r), c(n', r)) \ , \tag{5}$$

as shown in the article. The transcript-transcript distance was calculated as $D_{nn'} = 1 - C_{nn'}$ (high occurrence is equivalent to short distance between transcripts) and used as input to a hierarchical clustering with the Ward distance. The leaves were subsequently ordered in such a way that adjacent clusters had maximum similarity. The dendrogram with 27 leaves gave us an ordering of the transcripts [12] that was used for data interpretation. The number of leaves was determined as described in the article.

Clustering verification steps

In the second interactive step of the cluster analysis procedure we addressed some of the shortcomings of clustering. Clustering of all 5,930 detectable SGD annotated ORFs produced both biologically meaningful clusters, but also clusters arising from experimental artefacts, e.g. array outliers. Secondly, in some cases clusters were so similar in shape and in gene ontology over-representation [61] that they could be merged into one cluster. Thus, we used the following steps

1. All clusters obtained from the robust clustering with the Bayesian consensus mechanism were analysed as described in previous section.

2. Clusters that appeared to capture artefacts were discarded and biologically meaningful clusters were stored. In a few cases, adjacent clusters in the leaf ordered dendrogram captured similar shape and gene ontology over-representation, and subsequently they were merged.

3. In the last step, P_a-values for each ORF were calculated based on a single clustering run in which the clusters were initiated in the stored (and merged) clusters. ORFs which could not be assigned to a cluster with $P_a < 0.20$ were discarded and collected in a 'trash' cluster together with the discarded clusters from step 2.

Clustering with the Bayesian consensus mechanism resulted in 27 clusters (**Figure 1** and **Figure 2**). An initial analysis of the over-represented gene ontology categories suggested that clusters (1 and 2), (9 and 10), (11 and 12), (22 and 23), (24 and 25), and (26 and 27) represented the same biological information, and hence these clusters were merged. Out of the 21 remaining clusters, a total of 8 clusters were discarded (clusters 3–7, 14, 15 and 19) resulting in 13 clusters with a clear trend. 1,018 ORFs with $P_a > 0.20$ were removed, and hence the total number of ORFs were reduced to 2,535 (**Table 1**, **Additional data file 3**). The 13 clusters were finally renumbered and displayed with the 'trash' cluster.

Table 1: Summary of data reduction after the clustering verification steps. Numbers in the left column indicate ORFs removed from the final analysis.

Features		6,091
Absent ORFs	161	161
Present ORFs		5,930
Artefacts	1,366	
<80% confidence	1,018	2,384
Changed transcription		3,546

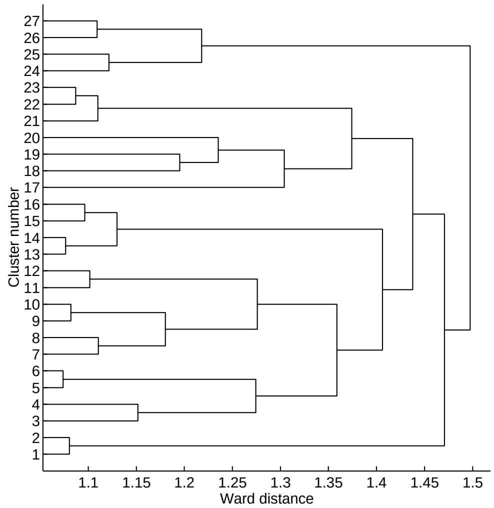

Figure 1: Dendrogram for leaf ordered hierarchical clustering of transcription data with the Ward distance used as metric. The expression profiles of the clusters are shown in Figure 2.

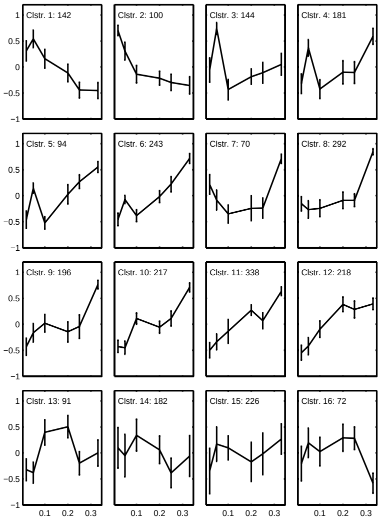

2.1: Clusters 1–16.

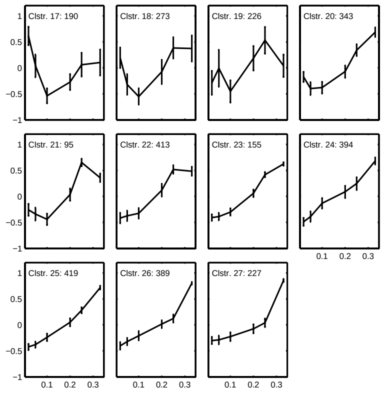

2.2: Clusters 17–27.

Figure 2: Overview of the original clusters. The transcript level for each transcript has been transformed to a value between -1 and 1, where 0 indicates the average expression level over all six growth conditions.
