# Supplementary material for: Growth-rate regulated genes have profound impact on interpretation of transcriptome profiling in Saccharomyces cerevisiae
Source: Genome Biol. 2006 Nov 14;7(11):R107. doi: 10.1186/gb-2006-7-11-r107 (PMC1794586; doi:10.1186/gb-2006-7-11-r107)
Supplement: Additional data file 4 — Document showing the influence of the number of replication origins on the P values when testing for correlation between genes and their location with respect to the replication origins. [file gb-2006-7-11-r107-S4.pdf]

Additional data file 4

The statistical significance between the position of replication origins and ORFs in each cluster was determined by randomisation tests. First, for all genes in a particular cluster, the average distance between the start codon (in base pairs) to the nearest of the 294 replication origins [34] was calculated. Second, the average distance for clusters with genes randomly distributed over all 16 chromosomes was repeatedly determined, and a P-value (the probability for observing the average distance in the cluster by chance) was calculated. The number of replication origins used in this study is much lower than the 429 replication origins determined by chromosome immunoprecipitation [32] and 332 found by replication timing experiment [33].

A sensitivity analysis of the result was done by decreasing the number of replication origins. The removed replication origins from the list of 294 were randomly selected multiple times in order to avoid a bias in the sensitivity analysis. In **Table 1** it is shown that a decrease between 0 and 80 replication origins results in a drastic increase in the P-value. Consequently, if the true number of replication origins is closer to the 429 replication origins determined by chromosome immunoprecipitation [32] a more significant correlation between replication origins and the down-regulated cluster 13 is expected.

Table 1: Sensitivity analysis of the correlation between replication origin and the down-regulated cluster 13. The result is given as P-values for the number of randomly removed replication origin in the consensus list of 294 replication origin.

Number of removed replication origins	0	20	40	80
P-value	0.0006	0.0030	0.0200	0.0300
