# Supplementary material for: Growth-rate regulated genes have profound impact on interpretation of transcriptome profiling in Saccharomyces cerevisiae
Source: Genome Biol. 2006 Nov 14;7(11):R107. doi: 10.1186/gb-2006-7-11-r107 (PMC1794586; doi:10.1186/gb-2006-7-11-r107)
Supplement: Additional data file 5 — Document describing the normalization with dChip and the subsequent comparison with a whole genome study with external RNA control as normalization reference. [file gb-2006-7-11-r107-S5.pdf]

Additional data file 5

All 12 DNA microarrays were scanned with the Affymetrix scanner, and the Affymetrix Microarray Suite v5.0 was used to generate CEL image files of the microarrays. The array images were then normalised with the software dChip v1.2, and the expression levels of all 9,335 probe sets were calculated with the Perfect Match (PM) model in dChip v1.2 assuming that the total mRNA level is constant for all specific growth rates [55]. This is the standard way of normalising data.

In a whole genome study with external RNA control as normalisation reference it was demonstrated that *S. cerevisiae* in stationary phase showed an 1.8 median ratio drop in the mRNA level compared to exponential growth [58]. However, 113 ORFs, without common biological functionality, had constant expression levels during both exponential growth and stationary phase (**Table 1**). Constant was here defined as ORFs where the logarithm to the median expression level was less than 0.10 (absolute level) and the logarithm to the highest value divided by the smallest value was less than 0.05.

Table 1: A list of 113 constantly expressed ORFs in exponential growth and stationary phase based on the study by van de Peppel et al. [58].

YAL001C	YAL028W	YAL063C	YBL067C	YBL095W	YBL098W	YBR019C
YBR145W	YBR204C	YCL032W	YCL033C	YCL056C	YDL022W	YDL069C
YDL110C	YDL123W	YDL233W	YDR014W	YDR039C	YDR055W	YDR082W
YDR104C	YDR179C	YDR202C	YDR286C	YDR293C	YDR306C	YDR379W
YDR384C	YDR401W	YEL008W	YER142C	YER173W	YFL016C	YFR014C
YFR027W	YGL005C	YGL053W	YGL056C	YGL154C	YGL185C	YGR010W
YGR102C	YGR133W	YGR203W	YGR244C	YGR268C	YHR034C	YHR075C
YHR080C	YHR090C	YHR105W	YHR195W	YIL139C	YIL152W	YJL137C
YJL164C	YJL222W	YKL067W	YKL159C	YKL220C	YKR067W	YLL006W
YLL027W	YLR097C	YLR102C	YLR219W	YLR228C	YLR266C	YLR298C
YLR306W	YLR323C	YLR352W	YLR408C	YML131W	YMR065W	YMR114C
YMR139W	YMR149W	YMR255W	YMR325W	YNL242W	YNL286W	YNL293W
YNR045W	YNR073C	YOL025W	YOL036W	YOL071W	YOL081W	YOL114C
YOR035C	YOR054C	YOR075W	YOR084W	YOR138C	YOR148C	YOR274W
YOR279C	YOR316C	YOR319W	YOR377W	YOR381W	YOR386W	YPL008W
YPL159C	YPL181W	YPL240C	YPR008W	YPR023C	YPR082C	YPR117W
YPR120C						

We analysed the transcription profile of these 113 ORFs in our study (standard normalisation) and observed that 88 ORFs were down-regulated with the specific growth rate. Moreover, 42 ORFs with an expression value above 500 were on average linearly down-regulated ($P < 0.05$) (**Table 2**). If the constraint about a negative slope was relaxed, an additional four genes with positive slope were included in the analysis: *YKL067W*, *YMR149W*, *YNR045W*, and *YFR014C*. Three out of these four genes were involved in macromolecule biosynthesis, and hence these discrepancies were probably due to different medium compositions between the two studies. The linear correlation between the relative transcript level Y (scaled such that $Y = 1$ at $\mu = 0$ for all ORFs) and the specific growth rate μ for all 42 ORFs with negative slopes was determined. The average, linear correlation was

$$Y = 1 - (1.6312 \pm 0.4264)\mu \tag{1}$$

where 0.4264 is the standard deviation. The correlation was not sensitive to choice of P-value and the minimum expression value of 500. At $\mu = 0^1/_h$ mRNA is not diluted (per definition), but e.g. at $\mu = 0.20^1/_h$ mRNA is diluted to 67% due to the normalisation assuming constant mRNA. Inserting $\mu = 0.02$ and $0.33^1/_\mathrm{h}$, growth rates approximating stationary phase and exponential growth, into **Equation 1** gives $Y_{0.02}/Y_{0.33} = 2.1 \simeq 1.8$ suggesting good agreement between the current study and the study by van de Peppel et al. [58]. Thus, in order

to compensate for this dilution effect the dataset was linearly scaled so the expression level of the 42 ORFs was constant.

Table 2: 42 ORFs from **Table 1** with an expression value above 500 were linearly down-regulated ($P < 0.05$) in our experiments.

YBL067C	YBR145W	YCL032W	YCL033C	YCL056C	YDL110C	YDR039C
YDR055W	YDR082W	YDR104C	YDR202C	YDR293C	YDR306C	YDR384C
YER142C	YER173W	YGL005C	YGL053W	YGL154C	YGL185C	YGR244C
YHR075C	YHR195W	YIL152W	YJL164C	YLR102C	YLR219W	YLR266C
YML131W	YMR139W	YNL293W	YOL025W	YOL071W	YOL081W	YOR035C
YOR054C	YOR148C	YOR279C	YOR319W	YPL159C	YPL240C	YPR008W
